# Heat Shock Response in CHO Mammalian Cells Is Controlled by a Nonlinear Stochastic Process

**DOI:** 10.1371/journal.pcbi.0030187

**Published:** 2007-10-05

**Authors:** Ovidiu Lipan, Jean-Marc Navenot, Zixuan Wang, Lei Huang, Stephen C Peiper

**Affiliations:** 1 Department of Physics, University of Richmond, Richmond, Virginia, United States of America; 2 Department of Pathology, Medical College of Georgia, Augusta, Georgia, United States of America; 3 Cancer Research Center, Medical College of Georgia, Augusta, Georgia, United States of America; 4 Immunotherapy Center, Medical College of Georgia, Augusta, Georgia, United States of America; 5 Department of Radiology, Medical College of Georgia, Augusta, Georgia, United States of America; 6 Center for Molecular Chaperone, Radiobiology, and Cancer Virology, Medical College of Georgia, Augusta, Georgia, United States of America; Lawrence Berkeley National Laboratory, United States of America

## Abstract

In many biological systems, the interactions that describe the coupling between different units in a genetic network are nonlinear and stochastic. We study the interplay between stochasticity and nonlinearity using the responses of Chinese hamster ovary (CHO) mammalian cells to different temperature shocks. The experimental data show that the mean value response of a cell population can be described by a mathematical expression (empirical law) which is valid for a large range of heat shock conditions. A nonlinear stochastic theoretical model was developed that explains the empirical law for the mean response. Moreover, the theoretical model predicts a specific biological probability distribution of responses for a cell population. The prediction was experimentally confirmed by measurements at the single-cell level. The computational approach can be used to study other nonlinear stochastic biological phenomena.

## Author Summary

The structure of an unknown biological system is uncovered by experimentally perturbing the system with a series of input signals. The response to these perturbations is measured as output signals. Then, the mathematical relation between the input and the output signals constitutes a model for the system. As a result, a classification of biological molecular networks can be devised using their input–output functional relation. This article studies the input–output functional form for the response to heat shocks in mammalian cells. The Chinese hamster ovary (CHO) mammalian cells were perturbed with a series of heat pulses of precise duration and temperature. The experimental data, taken at the single-cell level, revealed a simple and precise mathematical law for the time evolution of the heat shock response. Parameters of the mathematical law can be experimentally measured and can be used by heat shock biologists to classify the heat shock response in different experimental conditions. Since the response to heat shock is the outcome of a transcriptional factor control, it is highly probable that the empirical law is valid for other biological systems. The mathematical model explains not only the mean value of the response but also the time evolution of its probability distribution in a cell population.

## Introduction

Complex biological systems are built out of a huge number of components. These components are diverse: DNA sequence elements, mRNA, transcription factors, etc. The concentration of each component changes over time. One way to understand the functions of a complex biological system is to construct a quantitative model of the interactions present in the system. These interactions are usually nonlinear in terms of the concentrations of the components that participate in the interaction process. For example, the concentration of a dimer is proportional to the product of the concentrations of the molecules that dimerise. Besides being nonlinear, the interactions are also stochastic. The production process of a molecule is not deterministic, and it is governed by a probability rate of production. In what follows, a nonlinear stochastic model for the response to heat shocks in CHO mammalian cells will be developed. Heat stress is just one example of the many ways a molecular system can be perturbed. From a general perspective, the structure of a molecular system is uncovered by imposing different perturbations (input signals) on the system under study, and then the responses of the system (output signals) are measured. From the experimental collection of pairs of input–output signals, laws that describe the system can be uncovered. This is the fundamental idea in Systems and Synthetic Biology [1–5] and has long proved to be successful in the field of electronics. The input signals are applied through the use of signal generators [6–8]. An input signal that is easy to manipulate is a heat pulse, the parameters to change being the pulse temperature and its duration. Members of the stress protein family such as the heat shock protein 70 (HSP70) are highly responsive to temperature variations. This protein is a molecular chaperone and is a critical component of a complex genetic network that enables the organism to respond to deleterious effects of stress [9–11]. Since Hsp70 is thus an important regulator in a complex system, our goal was to find if it is possible to develop a mathematical model of the regulation of its expression in mammalian cells exposed to heat shock. Our specific objectives were 1) determine an equation representing the average expression of Hsp70 over time in a cell population after an initial heat shock, 2) determine how the physical parameters of heat shock (temperature and duration) influence the parameters of this equation, and 3) determine the mathematical model that describes the expression of Hsp70 at the single-cell level. We first describe the process of inferring the mathematical model from the experimental data. Then a mathematical study of the model will follow.

## Results

### The Double Exponential Response to Heat Shocks. From Experiment to a Theoretical Model

To acquire the experimental data, we elected to use a system using a reporter gene where the expression of the green fluorescent protein (GFP) is under the control of the promoter region of the mouse *Hsp70* gene. The GFP reporter proved useful for quantitative analysis [12] and was used before in connection with Hsp70 in different biological systems [13–17]. The *Hsp70-GFP* fusion gene was integrated into a plasmid and transfected in Chinese hamster ovary (CHO) cells. Stable transfectants were selected for their low level of basal expression of GFP and their capacity to upregulate GFP effectively and homogenously after exposure to heat shock. Flow cytometry was used to make precise quantitative measurements of the fluorescence of a large cell population. Since the quality of the experimental data was critical to the feasibility of the mathematical analysis, steps were taken to minimize sample-to-sample and experiment-to-experiment variability and to maintain the experimental noise to a minimum. To that effect, temperature and time were tightly controlled for heat shocks, the cells were treated as a batch in a single tube for each condition (combination of temperature and time), and aliquots were taken at each time point. All samples were fixed for at least 24 h before analysis by flow cytometry so that changes of fluorescence due to fixation would not be a factor, and all the samples from the same experiment were analyzed at the same time. Flow cytometry was chosen for analysis because it allows a very accurate quantitative measurement of the fluorescence of a large number of events, independently of the actual size of the sample. Within the same experiment and between experiments, the same instruments settings were used for the flow cytometer, and at least 1 × 10^4^ cells were analyzed per sample. Detailed protocols and experimental conditions are available in the Materials and Methods section.

**Figure 1:**
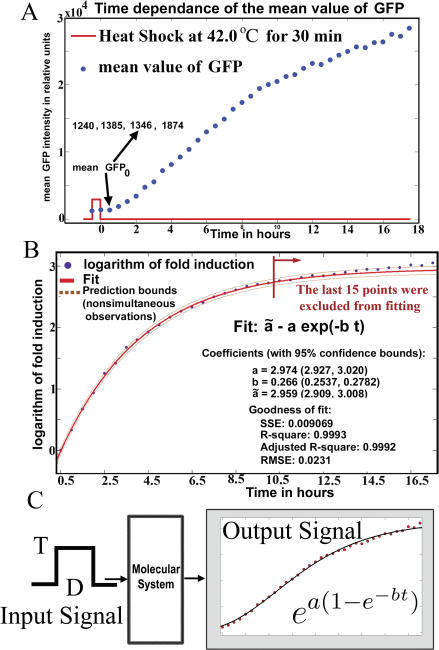
The Double Exponential Law (A) The accumulation of GFP is monitored for 18 h after heat shock. The fold induction is defined as the ratio of the mean value of GFP at different times (mean GFP) over the mean value at 30 min after the shock (mean GFP_0_). (B) The logarithm of the fold induction saturates exponentially in time. The last 15 samples were predicted by the fit on the first 25 points. (C) The formula 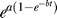 expresses the GFP fold induction as a function of time *t*. Parameters *a* and *b* are programmed into the systems' response during the duration of the heat pulse.

First, we will follow a description of the time course of the mean response to a heat shock. At elevated temperatures (39 °C to 47 °C), the heat shock promoter HSP70 is active and GFP starts to be synthesized. The input signals were chosen in the form of a pulse at a temperature (*T*) and duration in time (D) (Figure 1A). In the first experiment, the dynamic response of GFP after a heat pulse at 42 °C for 30 min was monitored by taking samples each 30 min for 18 h. Before and immediately after the heat shock, the GFP intensity remains at approximately the same level; this phenomenon was observed in all subsequent experiments.

The fold induction of GFP with respect to a reference (*GFP*_0_) was then determined:

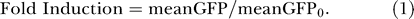



The reference is the first measured sample away from the end of the heat shock (30 min after the shock in Figure 1A). Our finding is that the logarithm of the fold induction of GFP follows an exponential saturation trajectory (Figure 1B), with tight confidence bounds for the estimated parameters and tight prediction bounds for nonsimultaneous observations. The tight prediction bounds appear even when almost half of the data is not used during fitting (Figure 1B).

The time *t* is measured relative to the reference time *t*_0_.The initial fold induction at *t* = 0 (or equivalent *t*_0_ after the end of heat shock) is 1. This value of 1 for the initial fold induction is consistent with the entire time evolution if a fit with the expression 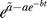 will give a value for parameter 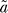 very close to the value for parameter *a*. Theoretically, 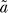 must be equal with *a* to have a fold induction of 1 at *t* = 0. The result of the fit (Figure 1B) shows this consistency. From now on we will take 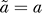 .

The empirical law for the response of the cells to the heat pulse can be thus cast into the form:

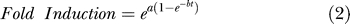



The same law appeared in repeated measurements of pulses at 42 °C for 30 min duration (unpublished data). Parameter *b* describes the quickness of the response. As *b* increases, the saturation value of the response is reached in less time. Parameter *a* specifies the saturation value of the response. The plateau reached by the fold induction is *e^a^* and thus grows exponentially with parameter *a*.

These findings suggest that the same law is valid for other heat shock pulses, parameters *a* and *b* being dependent on the heat pulse height *T* and its duration *D* (Figure 1C).

**Figure 2:**
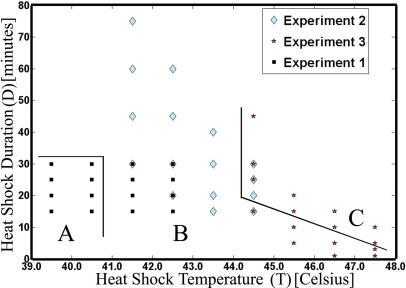
Sixteen Pairs of (T, D) Conditions Were Run in Each Experiment For each heat shock pulse (T, D), 13 time samples were taken. At each time sample, the intensity of GFP in at least 10,000 cells was recorded. The groups A, B, and C represent weak, moderate, and strong heat shocks, respectively.

To find the range of validity for the empirical law, measurements were taken for the responses to heat shocks at various heat pulse parameters *T* and *D* in a series of three experiments that partially overlapped Figure 2.

**Figure 3:**
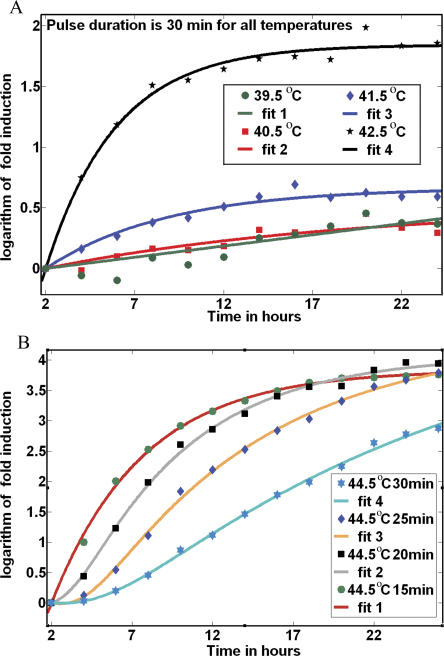
The Law Is the Same for All Temperatures (A) For weak shocks (39.5 °C to 40.5 °C), the fits are less tight than they are for moderate shocks (41.5 °C and 42.5 °C). (B) For strong heat shocks (duration greater than 15 min in this figure), the response starts at a slow pace. Later, the response grows faster, overcoming those responses produced by less strong shocks. The time origin and the reference value for fold induction, GFP_0_, is the mean response at 2 h after the shock.

The law was again present in all responses for temperatures between 41.5 °C and 42.5 °C, (examples selected in Figure 3A, fit 3, 4). For lower temperature (39.5 °C to 40.5 °C), the law was valid, but with poor 95% confidence intervals for estimated parameters *a* and *b*, as in Figure 3A, fit 1, 2 (the activity of the Hsp70 promoter was low). At high temperatures or long durations (Figure 3B), the double exponential law still explains the main characteristic of the stress response and is valid after a few hours from the end of the heat shock.

In the following, a theoretical model will be developed to explain the experimentally discovered law. The exponential accumulation of the GFP shows that the derivative with respect to time of the mean GFP is proportional with itself:

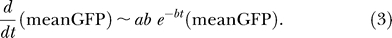



**Figure 4:**
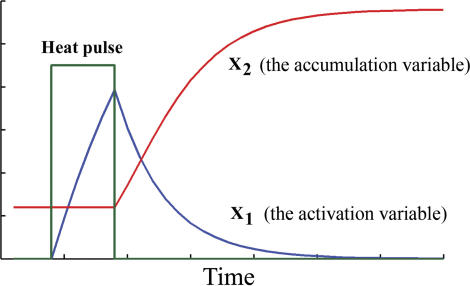
GFP is Proportional with *X*_2_ The accumulation rate of *X*_2_ is controlled by *X*_1_. For weak and moderate shocks, the activation component *X*_1_ reaches high values at the end of the heat pulse. The degradation rate of *X*_1_ after the heat pulse is proportional with parameter *b*. The accumulation height of *X*_2_ at the end of the heat pulse depends on parameter *a*.

There must be thus a molecular process, described by the exponential term *abe^−bt^*, which controls the heat shock response. This theoretical suggestion is confirmed by previous studies of the heat shock system which revealed that the accumulation and subsequent degradation of the heat shock transcription factor 1 (HSF1) regulates Hsp70 [18–22]. Experimental results [18] show that HSF1 activation is characterized by a rapid and transient increase in *hsp70* transcription which parallels the kinetics of HSF1–DNA binding and inducible phosphorylation. This rapid increase in HSF1–DNA binding activity reaches a maximal level and thereafter attenuates to a low level. This rapid increase in activity followed by attenuation will form the starting point for our theoretical model. An activation–accumulation two-component model will be developed as a minimal theoretical description of the empirical law. The “activation” variable (*X*_1_) represents the first phase of the heat shock response and includes components like HSF1–DNA binding activity. *X*_1_ will increase during the duration of the heat shock and then, after the shock, will decrease with a lifetime proportional to parameter *b* (Figure 4).

The “accumulation” variable (*X*_2_) includes the products of transcription and translation. This second variable, at low levels before the shock, will gain momentum after the shock. To connect the model with the experimental data, the GFP will be considered to be proportional with *X*_2_. The speed of accumulation of *X*_2_, that is, *dX*_2_/*dt*, will be proportional to the product *X*_1_ *X*_2_. Immediately after the shock, *X*_1_ has a big value (the activation is high), and thus the speed of *X*_2_ is high (the accumulation is in full thrust). This will trigger an initial fast accumulation of GFP, which is proportional with *X*_2_. Later on, the activity *X*_1_ disappears, nullifying the product *X*_1_ *X*_2_ and thus the speed of *X*_2_. The process is then terminated (the accumulation stops) (Figure 4). The empirical law follows directly as a solution of the activation–accumulation system of equations:

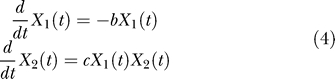
with *b* and *c* as some constants. Indeed, given the initial conditions *X*_1_(0) and *X*_2_(0) at a zero time reference *t*_0_ = 0, the solution to this system of differential equations is

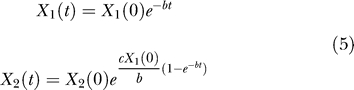
With the notation

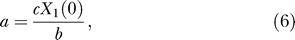
the empirical law follows from *X*_2_(*t*):

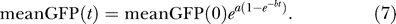



The theoretical model contains two parameters: *b* and *c*. Parameter *b* is directly accessible to experimental measurements, whereas parameter *c* is not; however, the product *cX*_1_(0) which equals the product of *a* and *b* can be measured.

It is interesting to notice that the above time evolution can be re-expressed as a conservation law which is independent of any reference time. For any two time points *t*_1_ and *t*_2_, the following holds

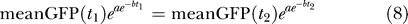



**Figure 5:**
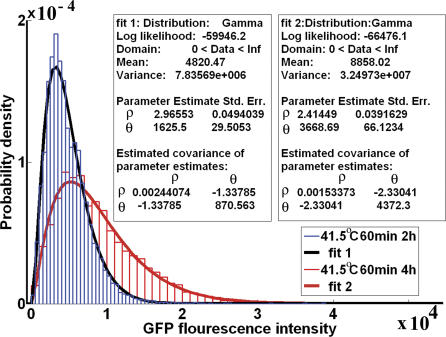
Experimental GFP Probability Density The experimental GFP fluorescence intensities are Gamma-distributed, as predicted by the activation–accumulation model.

At this point, there is no more information in the activation–accumulation description above than is in the empirical law. However, one can search for more information hidden in the above two-component description by turning attention to the full data available, not only to the mean value of GFP. For each sampled time, the full data available consists of measured GFP levels for at least 10,000 single cells. These 10,000 single-cell measurements are typically distributed as in Figure 5. There is a long tail at high values of GFP. This biological variation in response to the stress is explained by turning the deterministic two-component system into a stochastic two-component system [6,7]. The stochastic description must be completely enforced by ideas behind the deterministic two-component system. The stochastic model is simple. *X*_1_ is the mean value of a stochastic activation variable which will be denoted by *q*_1_, *X*_1_ = 〈*q*_1_〉. After the heat shock, *q*_1_ will decrease with a probabilistic transition rate *bq*_1_. The activation–accumulation stochastic model is based on the same relation as before (compare *bq*_1_ with *bX*_1_), but now it describes the probabilistic transition rate and not a deterministic speed of attenuation. By the same token, *X*_2_ is the mean value of *q*_2_ and its probabilistic accumulation rate is *cq*_1_ *q*_2_. One notices that the transition probability rate *cq*_1_ *q*_2_ is nonlinear in the variables *q*_1_ and *q*_2_. The stochastic two-component description is thus a mirror image of the deterministic two-component system. However, the probabilistic system is more powerful as it predicts that the histograms of GFP (proportional with *q*_2_) obtained from the flow cytometry measurements follow a Gamma distribution

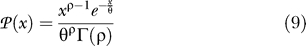
with GFP ≡ *x*. This prediction is confirmed experimentally (Figure 5).

**Figure 6:**
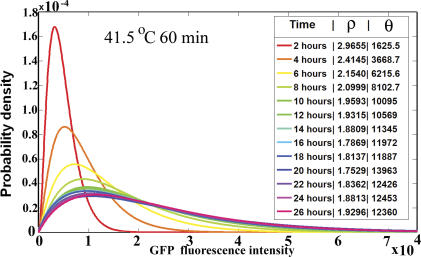
The Time Evolution of the Biological Variation in GFP As time develops, the biological heterogeneity increases. At all times, the heterogeneity is Gamma-distributed. Gamma distribution parameters *ρ* and *θ* are inserted in the legend.

The fact that the levels of proteins in gene networks tend to follow a Gamma distribution, which is a continuum version of a discrete negative-binomial distribution, was presented in [23,24]. The papers [23,24] develop theoretical models describing the steady-state distribution of protein concentration in live cells. Our interest lies in the non–steady-state behavior of these distributions. Namely, the aim is to find the time evolution of the parameters that characterize these distributions. The entire time evolution of the distributions is presented in Figure 6. The distributions become wider as time passes. The experimental data reveal that parameter *ρ* remains constant in time and only *θ* changes. These experimental findings are theoretically explained in detail in the section Analysis of the Theoretical Model. What follows summarizes the theoretical conclusions that are useful in understanding the experimental results of Figures 5 and 6. The probability distribution for the discrete molecule number *q*_2_, predicted by the stochastic activation–accumulation model, is the negative-binomial distribution. This distribution appeared in earlier theoretical studies of genetic networks [23,24] and in physics [25,26]. The GFP intensity is proportional with *q*_2_ and appears in measurements as a decimal number and not as a pure integer. Thus, to describe the probability distribution of the GFP intensity, a continuous version of the discrete negative-binomial distribution is necessary. This continuous version is the Gamma distribution observed experimentally in Figures 5 and 6. The physical interpretation of parameter *ρ* will now be discussed. At initial time *t*_0_, immediately after the heat shock, there will be at least one cell from the entire cell population which contains the minimum number of molecules *q*_2_. Denote this number by *N*_0_. As the time passes, the molecule number *q*_2_ will grow, following the described stochastic process. However, there is a nonzero probability, though extremely small, that the process of accumulation in one cell does not start even after 24 h. This can happen in one of those cells that contain the minimum number of molecules *q*_2_ at the initial time *t*_0_. Thus, at any later time *t* > *t*_0_, the lowest possible number of molecules *q*_2_ in a cell is *N*_0_ as it was at the initial time *t*_0_. It can be shown (see the section Analysis of the Theoretical Model) that *ρ* = *N*_0_. This explains the time independence of the experimental values of *ρ*; it also gives a physical meaning to *ρ* as being proportional to the minimum number of GFP molecules in a cell. Parameter *θ* contains the time evolution of the stochastic accumulation of the GFP molecules. This evolution can be again expressed as a time conservation property

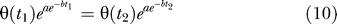
valid between any two time points *t*_1_ and *t*_2_. The above relation Equation 10 contains parameters *a* and *b* and can be used to check the consistency of the model. Using the data from Figure 6, it follows that *a* = 3.159 with a 95% confidence interval (3.074, 3.244) and *b* = 0.2572 with a 95% confidence interval (0.2358, 0.2785). From the mean value for GFP, it results that *a* = 2.423 with a 95% confidence interval (2.351, 2.496) and *b* = 0.2579 with a 95% confidence interval (0.2344, 0.2814). Parameter *a* is sensitive to the estimation procedure, a phenomenon connected with the fact that parameter *ρ* is not perfectly constant but decreases a bit with time. The mean value of the Gamma distribution is *ρθ.* For a perfectly constant *ρ*, the estimated value for *a* would be the same using either the *θ* values or the *ρθ* data. Contrary to parameter *a*, parameter *b* is independent of the way it is estimated, and the estimation is highly reliable.

**Figure 7:**
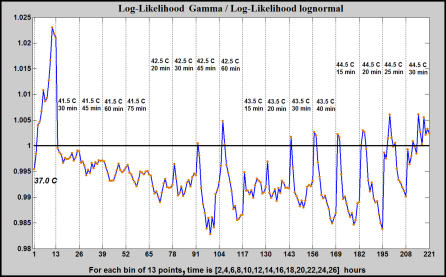
The Log-Likelihood Ratio of Gamma and Lognormal Distributions Depend on the Heat Shock Parameters For 37 °C, the lognormal fits data better than the Gamma distribution. As the heat shock is increased from low to moderate, the Gamma distribution becomes a better fit. For strong heat shocks (at 44.5 °C for 30 min), there is no a clear separation between a Gamma distribution and a lognormal one.

To further check the reality of the Gamma distribution for heat shock response, a comparison of the Gamma fit with the lognormal fit is presented in Figure 7. The lognormal was chosen because it can be viewed as a result of many random multiplicative biological processes. A loglikelihood ratio less than 1 favors the Gamma distribution against the lognormal. Moreover, at 37 °C the Gamma distribution is not a good fit (loglikelihood ratio is bigger than 1) as it should be because the promoter is not active.

**Figure 8:**
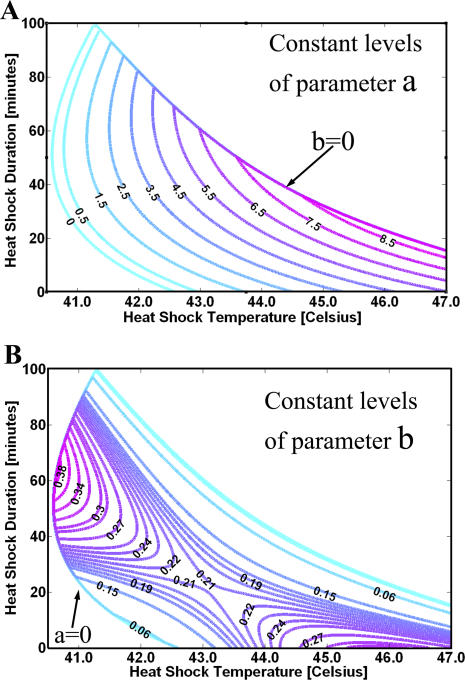
Variation of Parameters *a* and *b* with Temperature and Duration of the Heat Pulse (A) The contours for *a* stop when *b* = 0 in the upper right region. (B) In the lower left region, the contours for *b* stop when *a* = 0. Around 43.0 °C and duration 22 min, parameter *b* = 0.21 h^−1^. The instability of the saddle configuration can be related to a need for sensitivity with variation in temperature and duration of the stress. Because of the double exponential law, a small variation in *b* will cause a big effect in the response to stress.

The law 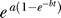 is useful in making predictions for the fold induction to many other heat shock pulses. For a heat pulse of a given temperature and duration, parameters *a* and *b* can be read out from Figure 8. The constant level contours were inferred from the experimental data. The level patterns differ; parameter *a* increases monotonically with the temperature and duration of the heat pulse (Figure 8A), while the levels of parameter *b* form an unstable saddle shape pattern (Figure 8B).

The conclusion of this section will be rephrased using a control theory perspective. The end result of this paper is an input–output relation for the response of the CHO cells to heat shocks, together with a theoretical model that explains it. The input signals are pulses of a precise time duration *D* and temperature height *T*. The output measured signals are the GFP intensity. The input–output relation is given by the time-dependent probability density for GFP intensity

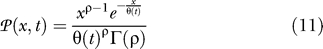
with

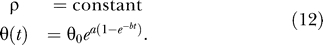



Parameters *a* and *b* are functions of the input signal, that is *a* = *a*(*T*, *D*) and *b* = *b*(*T*, *D*). The dependence of parameters *a* and *b* on temperature *T* and duration *D* is given by the contour plots of Figure 8. The functional forms of *a* = *a*(*T*, *D*) and *b* = *b*(*T*, *D*) is a consequence of biological phenomena that take place during the heat shock. We do not have a theoretical model for the phenomena that take place during the heat shock. To explain the time evolution of the output variable (GFP intensity), we developed a coarse-grained model for the heat shock response. This coarse-grained model is valid for the biological phenomena that takes place after the end of the heat shock. The model predicts the existence of a molecular factor that controls the GFP accumulation (variable *q*_1_). We associated this theoretical factor with the heat shock factor HSF1-DNA binding activity.

### Analysis of the Theoretical Model

**Figure 9:**
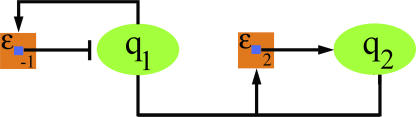
Molecular Diagram for the Activation–Accumulation Process

The theoretical model is based on an activation variable *q*_1_ and an accumulation variable *q*_2_. The state of this two-component model is thus (*q*_1_, *q*_2_), and any pair of positive integer numbers can be a possible state. The main goal is to find the mean value and standard deviation for the activation and accumulation variable, respectively. These quantities will be obtained from the equation for the probability *P*(*q*_1_, *q*_2_, *t*) that the system is in the state (*q*_1_, *q*_2_) at the time *t*. The equation for *P*(*q*_1_, *q*_2_, *t*) depends on the multitude of transitions which can change a state (*q*_1_, *q*_2_). The experimental results suggest that two possible transitions change the state (*q*_1_, *q*_2_). One transition represents the decreasing of the activation variable from *q*_1_ to *q*_1_ − 1. On the state (*q*_1_, *q*_2_), this attenuation appears as (*q*_1_, *q*_2_) → (*q*_1_ − 1, *q*_2_), with an unaffected accumulation variable *q*_2_. The second transition will describe the accumulation of the accumulation variable from *q*_2_ to *q*_2_ + 1. On the state (*q*_1_, *q*_2_), this accumulation appears as (*q*_1_, *q*_2_) → (*q*_1_, *q*_2_ + 1), with the activation variable *q*_1_ now being unaffected. A notation for the transition direction can be introduced: *ɛ*_−1_ = (−1,0). The degradation transition can thus be written as (*q*_1_, *q*_2_) → (*q*_1_, *q*_2_) + *ɛ*_−1_. The negative sign in the index −1 is just a reminder of the fact that the transition reduces the number of molecules; the 1 in the subscript tells us that the transition is on the first variable. Likewise, the accumulation transition can be expressed as (*q*_1_, *q*_2_) → (*q*_1_, *q*_2_) + *ɛ*_2_ and *ɛ*_2_ = (0,1). The index 2 is positive (accumulation) and is associated with the second component. To find the probability *P*(*q*_1_, *q*_2_, *t*), the transition probabilities per unit time are needed. The experiment suggests we use

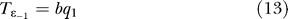
as the transition probability rate for the attenuation of the activation component, and

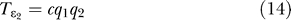
as the transition probability rate for the increasing of the accumulation component. The stochastic model can be represented with the help of a molecular diagram [7] (Figure 9).

The components *q*_1_ and *q*_2_ are represented by ovals and the transitions by squares. The lines that start from the center of a transition square represent the sign of that transition and point to the component on which the transition acts. The transition *ɛ*_−1_ is negative, so the line ends in a bar and acts on *q*_1_. The transition *ɛ*_2_ is positive and so the line ends with an arrow; it acts on *q*_2_. The lines that stop on the edges of the transition squares represent the transition probability rates. The line that starts from *q*_1_ and ends on *ɛ*_−1_ represents the transition probability rate *bq*_1_. In other words, the transition *ɛ*_−1_ is controlled by *q*_1_. The lines that start on *q*_1_ and *q*_2_ and merge together to end on *ɛ*_2_ represent the product *cq*_1_ *q*_2_, (the merging point represents the mathematical operation of taking the product).

At this point, the theoretical model is fixed and what comes next is a sequence of computations to extract information out of it. This information will be compared with the experimental results. Given the transition rates, the equation for the probability *P*(*q*_1_,*q*_2_,*t*) is given by the following equation [7,25].

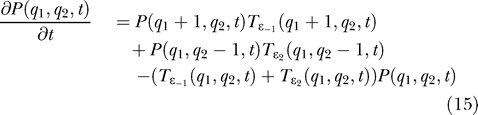



The above equation for *P*(*q*_1_,*q*_2_,*t*) is not easy to solve. We will use the method outlined in [6,7] and work with the function *X*(*z*_1_,*z*_2_,*t*) defined by

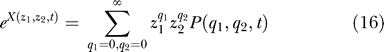



The equation for the function *X*(*z*_1_,*z*_2_,*t*) is a consequence of the equation for *P*(*q*_1_,*q*_2_,*t*):

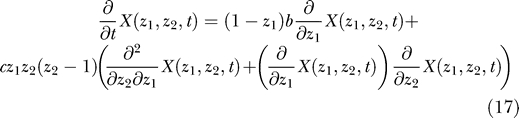



The goal is to find the time variation of the mean value and standard deviation for the activation and accumulation variable: 〈*q*_1_〉, 〈*q*_2_〉, 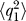 , 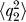 , 〈*q*_1_,*q*_2_〉, etc. Here 〈〉 is a notation for the mean value with respect to the probability distribution *P*(*q*_1_,*q*_2_,*t*). From *X*(*z*_1_,*z*_2_,*t*), the above mean values can be obtained by taking partial derivatives of *X*(*z*_1_,*z*_2_,*t*) at *z*_1_ =1, *z*_2_ = 1. These partial derivatives are actually the *factorial cumulants* of the probability distribution *P*(*q*_1_,*q*_2_,*t*). In what follows, the sign =: means that the right side is introduced as a notation.

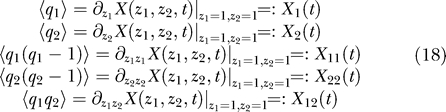



The equations for *X*_1_(*t*), *X*_2_(*t*), *X*_11_(*t*), and higher factorial cumulants result from the equation for *X*(*z*_1_,*z*_2_,*t*):

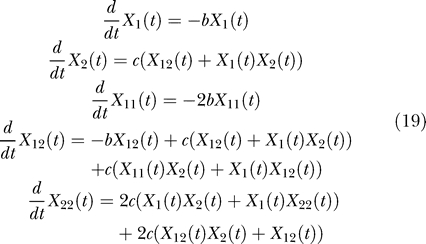



The activation–accumulation model being nonlinear, the equations for the factorial cumulants cannot be reduced to a finite system of equations, unless some approximation technique is employed. All third-order cumulants were discarded to obtain the above system of equations. In [7] it was shown, using simulations, that the effect of discarding higher-order factorial cumulants is negligible. The finite system thus obtained contains *X*_1_, *X*_2_, *X*_12_, *X*_11_, and *X*_22_ as variables. Although it can be solved for *X*_1_ and *X*_2_, we found that the influence of the correlation term *X*_12_ is small and cannot be experimentally detected in the GFP response. Taken thus, *X*_12_ = 0, and the system of equations is reduced to:

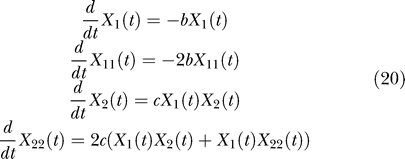



The solution to *X*_22_ from the four-equation system is

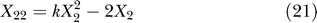
with *k* a constant determined from the initial value *X*_2_(*t*_0_) at some time *t*_0_ after the heat shock. The solution can be restated in terms of the variance, Var, of the variable *q*_2_. The transformation from the factorial cumulants to Var is

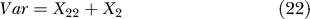
And, thus, remembering that the mean value of *q*_2_ is *X*_22_, it follows that

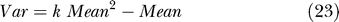
Such a relation between *Var* and *Mean* is satisfied by the negative-binomial distribution, a point to which we will return later. Employing the general procedure, we continue to solve the system of equations for *X*_1_, *X*_2_, *X*_11_, and *X*_22_. However, for the case of negligible *X*_12_, the stochastic process is decoupled in two stochastic processes, each of which is exactly solvable. It is thus useful to solve directly for the probability distribution of *q*_2_ at this point. The transition probability rate for the first stochastic process (for the activation component *q*_1_) is the same as before: 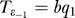 . For the second one, it changes from 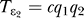 to 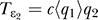 (the coupling between *q*_1_ and *q*_2_ is through the *mean* value of *q*_1_ now). This simplifies the problem of finding the distribution of *q*_2_. Denote the mean value of *cq*_1_ with *g*(*t*), which acts actually like a *signal generator* on *q*_2_ [6,7]. The time variation of *g*(*t*) from the first equation in Equation 20 is

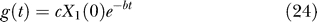
so the stochastic process for *q*_2_ now has an accumulation transition rate

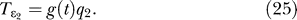



The origin of time, *t* = 0, is taken at the end of the heat shock, so *X*_1_(0) represents the mean value of the activation variable at the end of the heat shock.

The probability *P*(*q*_2_,*t*) to have *q*_2_ number of molecules at time *t* can be found from the master equation for this process

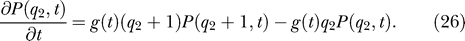



To find the solution, an initial condition *P*(*q*_2_,*t*_0_) must be specified. The time *t* = *t*_0_ is some time taken after the heat shock pulse (*t*_0_ > 0), when the effects of the shock start to be detectable; it can be, for example, 30 min or 2 h after the pulse. The probability distribution *P*(*q*_2_,*t*_0_) can be obtained, in principle, from the experimental values of GFP since GFP = *fq*_2_. There is an obstacle though: the proportionality factor *f* is unknown. The factor *f* converts the number of molecules *q*_2_ into the laser intensity which is the output of the flow cytometry machine. The conversion from the molecule numbers to the laser intensity can be more complicated than the proportionality relation GFP = *fq*_2_. For example, a background *B* can change the relation into: GFP = *fq*_2_ + *B*. We measured the GFP in regular CHO cells (no *HFP70-GFP* construct) and found that the background *B* is about 50 times less than the minimum intensity of GFP in the transfected CHO cells. The settings of the flow cytometry instrument were set in a linear response range, and thus we will use the scaling relation GFP = *fq*_2_ to connect the flow cytometry readings with the number of molecules. To conclude this initial condition discussion, in a perfect setting we would know the scaling factor *f* and then get *P*(*q*_2_,*t*_0_) from the measured data. Because the scaling factor *f* is unknown, the problem will be solved in two steps. The first step in choosing *P*(*q*_2_,*t*_0_) is based on a simple assumption: all cells have the same number of molecules *q*_2_ = N at the time *t* = *t*_0_. That is *P*(*q*_2_,*t*_0_) = *δ*(*q*_2_,N) where *δ* is the Kronecker delta function. The solution to Equation 26 with this initial condition is

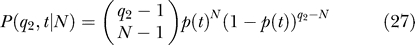



Here *q*_2_ can take only values greater than N, *q*_2_ = *N*, *N* + 1, ···. This distribution appeared in the study of cosmic rays [26], and in the context of protein production was presented in [23]. In terms of the variable *x* = *q*_2_ − *N*, it is known as the negative-binomial distribution, with interpretations that are not connected with the present problem. The number *N* also represents the minimum possible number of molecules *q*_2_ in any cell. This physical interpretation of *N* will be helpful in what follows. The variable *p*(*t*) in the distribution is time-dependent, since the signal generator *g*(*t*) acts on *q*_2_:

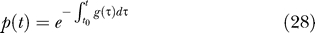



The mean and variance for *q*_2_ are

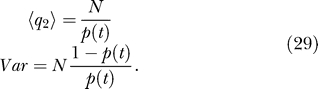
from which follows Equation 23

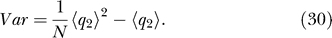



Although the assumption that all the cells contain the same number of molecules at *t* = *t*_0_ is unreal, it produces a valuable outcome. The negative-binomial distribution implies a Gamma distribution for the GFP intensity (through the scaling relation GFP = *fq*_2_), a fact to be discussed shortly. Because the Gamma distribution is a good fit for the experimental data, we conclude that the negative-binomial is the correct solution for the distribution of the accumulation variable *q*_2_.

The second step in choosing the probability distribution *P*(*q*_2_, *t*_0_) will be guided by the experimental results. The experimental results show that the biological system passes through a chain of events from an unknown distribution of GFP before the heat shock, to a Gamma distribution at some time *t*_0_ after the heat shock (2 h, for example). Also, the experiment shows that the distribution of GFP is Gamma at later times *t* > *t*_0_. In other words, the distribution of *q*_2_ becomes a negative-binomial at some time *t*_0_ after the heat shock and then afterward remains negative-binomial. These experimental observations are mathematically explained by showing that a solution to Equation 26 with a negative-binomial distribution at *t*_0_ remains negative-binomial for all later times *t* > *t*_0_. Indeed, the solution to Equation 26 with a negative-binomial initial condition

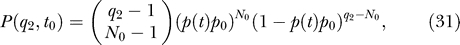
is

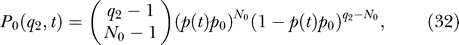
which is a negative-binomial at all times *t* > *t*_0_.

The number *N*_0_ is the minimum number of molecules *q*_2_ to be found in a cell at *t*_0_ and also at all later times *t* > *t*_0_ (because *q*_2_ cannot decrease).

The time evolution of the mean 〈*q*_2_〉 is

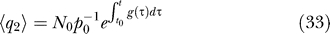
and represents, using Equation 24, the same empirical law (Equation 8) as before. To conclude, the dynamical system is such that once the cells enter into a negative-binomial distribution at some time after the heat shock, the distribution remains negative-binomial at later times. As the time passes, all the distributions will have the same parameter *N*_0_ but different parameters *p*(*t*).

To connect the theory with the experimental results, the probability distribution for the GFP intensity is needed. This distribution is the continuum limit of the distribution for *q*_2_. It is a well-known fact that the continuum limit of a negative-binomial distribution is the Gamma distribution. This continuum limit is presented here in order to find parameters *ρ* and *θ*, which can be experimentally measured.

The change from the integer variable *q*_2_ to the real variable *fq*_2_ is simple if advantage is taken of the fact that the common parameter *N*_0_ is a small number. Parameter *N*_0_ is less than any possible molecule number *q*_2_ present in the system after the time *t*_0_, *q*_2_ ≫ *N*_0_. Then, writing for simplicity *p*(*t*) as *p*,

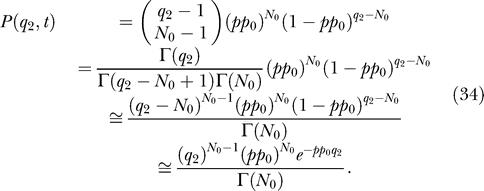



In the last step, we used the approximation 1 − *y* ≅ *e* ^−y^ for small values of *y*.

To go from the discrete variable *q*_2_ to the continuous variable GFP, we write the above relation as an equation for the probability density

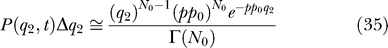
with Δ*q*_2_ = 1; then scale to GFP, (GFP = *f q*_2_). The probability density P℘ for GFP is then

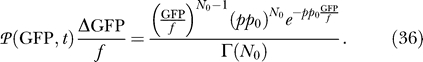



This is a Gamma distribution for GFP ≡ *x*

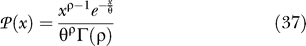
with

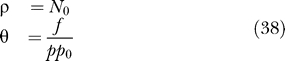
From Equation 24 and 28, we get

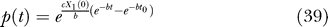



The mean value of the Gamma distribution is *ρθ* from which the empirical law Equation 8 follows.

## Discussion

The way the material is organized and presented in this paper is an outcome of a series of guiding principles imposed upon the project. These guiding principles were formulated to keep in balance the experimental data with both the mathematical and biological models. The guiding principles are: 1) start from experimental measurements and discover an empirical law from data using signal generators as input into the system; 2) build a simple mathematical model with as few parameters as possible to explain the empirical law; 3) check the mathematical model using additional experimental information; 4) use a general mathematical technique, likely to be applied to other experimental designs; 5) keep the biological model and the mathematical model to a level of complexity commensurate with the richness of the experimental data

These guiding principles filtered out other possible presentation formats. For example, the fifth principle will prevent the development of a complex mathematical model built on a complex biological model, although many molecules involved in the heat shock response are known. One outcome of the strategy outlined above is the discovery of a new variable, *q*_1_, brought about by a mathematical necessity from the empirical law. The behavior of this variable matches the behavior of the HSF1-DNA binding activity, experimentally described in [18]. In other words the empirical law predicts the existence of a heat shock factor, a molecule on which we did not take any measurements. This heat shock factor is well-known, but our point is that in other experimental settings a mathematical analysis of an empirical law can suggest the existence of an unknown molecule. In this respect, the aim of finding empirical laws from experimental data has biological significance. From a different perspective, an empirical law can be useful for calibration or classification purposes. For example, the heat shock responses can be classified using two parameters, *a* and *b*. Reproducibility of experiments can be checked by measuring parameter *b* which proved to be reproducible and reliable. Comparisons between different heat shock experiments can be done in terms of parameters *a* and *b*.

At a deeper level, the double exponential law and the activation–accumulation model need to be extended by simultaneously measuring the GFP production and the HSF1 activity. Following a series of modelling and data acquisition, more and more molecules can be reliably added into a quantitative description of the heat shock response.

Narrowing the discussion from general views to the specifics of this project, a natural question arises: why would cells evolve such a double exponential response? We can only speculate and say that cells need a very fast response immediately after the shock. Moreover, cells cannot bear for a long time such a fast exponential accumulation, so this initial exponential growth must be stopped. A compromise between these two requirements is the double exponential law for the mean heat shock response 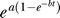 . Such a law permits a fast response immediately after the stress, controlled by parameter *a* and a flexibility in the duration of the response, controlled by parameter *b*. Such a response is easily implemented by a heat shock factor with an exponentially diminishing activity. We believe that this type of response is present in many other biological systems and thus has a wide range of applicability.

Another aspect to be noted is the time evolution of the stochastic process that describes the heat shock response. Not only the time evolution of the mean value can be mathematically modelled, but also the time evolution of the probability distribution.

The time evolution of GFP distribution can be well-explained by a negative-binomial with a time-dependent parameter. This behavior is obtained by neglecting the statistical correlation between the activation and the accumulation variable in the stochastic activation–accumulation model. It will be interesting to reach a level of experimental accuracy at which the statistical correlation becomes detectable, and then measure the deviation of the probability distributions from the negative-binomial.

From a mathematical point of view, we choose to work with the discrete master equation because it is simple to relate it to a biological model. The transition probability rates can be easily connected with biological phenomena at the molecular level. The ease of building the model is counterweighed by the difficulty of solving the discrete master equation. To overcome this difficulty, we employ the method outlined in [7], which uses the factorial cumulants as time-dependent variables.

The biological significance of the approach can be also expressed using a control theory perspective. The structure of an unknown physical system is uncovered by perturbing the system with a series of input signals. The response to these perturbations is measured as output signals. Then the mathematical relation between the input and the output signals constitutes a model for the system. As much as possible, this theoretical model must also incorporate the molecular components of the system. The activation–accumulation model belongs to the category of input–output models. It is possible that other biological systems can be described by other simple models. A classification of molecular networks can thus be devised using their input–output functional relation. Moreover, decomposing the biological system in subsystems, there is a hope that global properties of each subsystem can also be described by a coarse-grained model. In this way, a hierarchy of models can be built to explain more and more details of a complex system.

## Materials and Methods

### Plasmid construction.

A 5.3-kilobase DNA containing promoter and 5′-untranslated region of the mouse *hsp70.1*gene was subcloned from a lambda phage clone carrying an *hsp70.1* gene identified by genomic library screening (Stratagene) using a human *hsp70.1* cDNA as a probe. A cDNA coding for the GFP with a polyA signal from SV40 large T antigen gene was engineered to fuse to the start codon (ATG) of the *hsp70.1* gene. The chimera gene was inserted into a pSP72 vector containing a hygromycin resistance gene in order to select for stable transfectants.

### Preparation of transfectants.

CHO-K1 cells (ATCC) were grown in MEM-alpha (Cellgro) containing penicillin, streptomycin, and amphotericin (Cellgro) and complemented with 10% FBS (Gemini Bio-Products). Cells were transfected by lipofection using Lipofectamine (Invitrogen) as previously described. After 10 d of selection in hygromycin (500 *μ*g/mL), single-cell clones were derived by limiting dilution. The screening was performed by epifluorescence (Nikon TE2000E), and clones with a low basal fluorescence intensity were selected and amplified for additional testing by flow cytometry. One clone with a low basal expression of GFP and the capacity to effectively and homogenously upregulate the expression of GFP after being submitted to heat shock (42 °C, 30 min) was selected to conduct all the subsequent experiments.

### Heat shock.

The cells were detached with trypsin and allowed to recover in suspension in complete growth medium for 3 to 4 h at 1 × 10^6^ cells/mL at 37 °C in a CO_2_ incubator. The cells were then aliquoted in 50 mL conical tubes, one for each experimental condition (temperature and duration of heat shock). Up to five different temperatures were tested simultaneously, one water-bath being used for each temperature. The temperature of each water-bath was accurately monitored with a precision Hg thermometer (accuracy ±0.1 °C). Then the cells were centrifuged, the medium was aspirated, and the heat was initiated by resuspending the cell pellet quickly at 5 × 10^5^ cells/mL in a medium prewarmed at the temperature selected for the heat shock. The tube was then placed in the same water-bath for the remainder of the heat shock, after which the tube was placed in ice-cold water and agitated for the amount of time that had previously been determined to be necessary to bring the temperature back to 37 °C (from 2 to 14 s). The tube containing the cells was then placed in a waterbath set at 37 °C. From that point on, samples were taken every 30 min or every 2 h for up to 26 h. In all experiments, a control where the cells were kept at 37 °C for the whole time was included. The exact duration of each heat shock was monitored with a stopwatch. This protocol allowed a very strict control over the amount of input applied to the cells. The cells were kept in suspension in the 50 mL tubes in a CO_2_ incubator at 37 °C for the rest of the experiment.

### Sample processing.

At each time point, 1 mL of cell suspension was removed from each tube and placed in a 5 mL tube. The cells were centrifuged for 2 min at 300 g, the supernatant was aspirated, and the cell pellet was resuspended in 500 *μ*L of fixation solution (PBS containing 1% paraformaldehyde) and kept at room temperature and in the dark until analysis. Since fixation can decrease the fluorescence intensity of GFP, the samples were analyzed at least 24 h after collection of the last time point, so that the duration of fixation would not introduce any artifact.

### Sample analysis.

The samples were analyzed by flow cytometry on an LSR II (Becton-Dickinson) equipped with a 488 nm solid state laser. The performance of the system was routinely checked with fluorescent beads (8-peak beads, Shero Rainbow, Spherotech), and the same instrument settings were used in all experiments, yielding almost identical fluorescence intensities every time for the cells kept at 37 °C. The cells were gated based on their forward scatter (FSC) and side scatter (SSC), and the same gate was used for all the samples. The fluorescence of each cell was measured based on the area of the corresponding pulse. The data were analyzed with the Diva software (Becton-Dickinson) for the mean fluorescence. The flow cytometry binary FCS files were converted to an ASCII text format with FCSExtract utility (Stowers Institute for Medical Research). The data were consequently analyzed with cftool and dfittool from MATLAB (MathWorks).

### Estimation of parameters *a* and *b*.

The time evolution of the mean GFP expressed with respect to a reference initial time *t*_0_ = 0 is

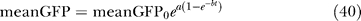



The above time evolution can be reexpressed as a conservation law which is independent of any reference time. For any two time points *t*_1_ and *t*_2_ we have

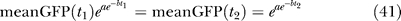
Taking thus a time reference *t*_0_ we get

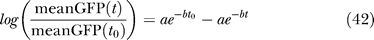
The form Equation 40 was used to estimate parameters *a* and *b* for medium and low heat shocks. For strong shocks we need to modify the estimation procedure. The reason for this modification is explained below.

As the promoter is activated by increasing temperature pulses, 41.5 °C to 43.5 °C, the Gamma distribution becomes a better description of the biological variation (Figure 7). However, for strong shocks, when the temperature approaches 44.5 °C and the duration of the pulse is high (30 min), the empirical law changes (Figures 7 and 3B). In the first hours after the shock, the response for a shock at 44.5 °C for 30 min is slower in comparison with a shock at 44.5 °C for 15 min. To account for this initial slow response, the experimental data for strong shocks call for a modification to the empirical law 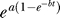 , valid at low and moderate shocks. For strong shocks, the modified empirical law that fit well the experimental mean response value is 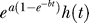 , where *h*(*t*) = 1 – *ξ* + *ξe* ^−*ηt*^. A few hours after the heat shock, when the effect of the exponential *e^−ηt^* is negligible and the slow response ended, the cell responds again with the same pattern found for low and moderate shocks.

The response at strong shocks can also be explained with the help of the activation–accumulation two-component model, by the following scenario. At the beginning of the heat shock, the activation component *X*_1_ will start to accumulate irrespective of how strong the heat shock will be. A cell does not know at the beginning of the heat shock about the duration of the shock. For a high temperature, if the duration of the shock is too long, after an initial accumulation, the activation component *X*_1_ will drop to low values. At the end of a strong shock, the activation component *X*_1_ will thus have low values. This is contrary to the case of moderate shocks, when at the end of the shock *X*_1_ has high values (Figure 4). This effect was observed experimentally in HeLa cells exposed to a 42 °C heat shock for 4 h [18]. The HSF1-DNA binding activity reaches its maximal level after 60 min and then attenuates to low levels at the end of the heat shock [18]. For strong shocks then, the activity *X*_1_ will accumulate again after the shock. Because *X*_1_ accumulates after the end of a strong shock, the speed of *X*_1_ is no longer described by *d*(*X*_1_)/*dt* = −*bX*_1_ in this time interval. Then, a few hours after the shock, it reaches a maximum value, from which it will decrease in the subsequent hours following *d*(*X*_1_)/*dt* = −*bX*_1_. The accumulation of *X*_1_ after the shock is responsible for the slow response in the first hours. The decrease of *X*_1_, which follows, imposes a response similar with the one observed at low and moderate shocks. The mathematical model for strong shocks during the time period when *X*_1_ decreases (5–6 h after the shock) is the same with the model for moderate shocks, *d*(*X*_1_)/*dt* = −*bX*_1_ and *d*(*X*_2_)/*dt* = *cX*_1_ *X*_2._. After the slow response ends, the empirical law 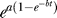 again explains the GFP trend.

In view of the above discussion, for strong shocks the mean GFP is given by a modification of Equation 40:

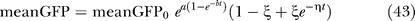
Similar to Equation 42, to estimate parameters *a,b*,*ξ*,*η* for strong shocks, we used

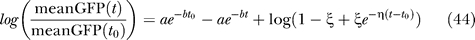



## Abbreviations

CHO: Chinese hamster ovary
GFP: green fluorescent protein
